# Biosynthesis of S-Adenosylmethionine by Magnetically Immobilized *Escherichia coli* Cells Highly Expressing a Methionine Adenosyltransferase Variant

**DOI:** 10.3390/molecules22081365

**Published:** 2017-08-18

**Authors:** Chunli Yin, Tao Zheng, Xin Chang

**Affiliations:** School of Biological and Environmental Engineering, Xi’an University, Xi’an 710065, China; zhengtaoangelov@126.com (T.Z.); newzealand233@163.com (X.C.)

**Keywords:** S-adenosylmethionine, hole-cell catalysis, methionine adenosyltransferase, magnetic immobilization, product inhibition, enzyme variants

## Abstract

S-Adenosylmethionine (SAM) is a natural metabolite having important uses in the treatment of various diseases. To develop a simple and effective way to produce SAM, immobilized *Escherichia coli* cells highly expressing an engineered variant of methionine adenosyltransferase (MAT) were employed to synthesize SAM. The recombinant I303V MAT variant was successfully produced at approximately 900 mg/L in a 10-L bioreactor and exhibited significantly less product inhibition and had a four-fold higher specific activity (14.2 U/mg) than the wild-type MAT (3.6 U/mg). To reduce the mass transfer resistance, the free whole-cells were permeabilized and immobilized using gellan gum gel as support in the presence of 100 mg/L Fe_3_O_4_ nanoparticles, and the highest activity (4152.4 U/L support) was obtained, with 78.2% of the activity recovery. The immobilized cells were more stable than the free cells under non-reactive conditions, with a half-life of 9.1 h at 50 °C. Furthermore, the magnetically immobilized cells were employed to produce SAM at a 40-mM scale. The residual activity of the immobilized cells was 67% of its initial activity after 10 reuses, and the conversion rate of ATP was ≥95% in all 10 batches. These results indicated that magnetically immobilized cells should be a promising biocatalyst for the biosynthesis of SAM.

## 1. Introduction

The primary metabolite S-adenosylmethionine (SAM), which is synthesized in vivo from l-methionine (l-Met) and adenosine triphosphate (ATP) by methionine adenosyltransferase (MAT), plays important roles in transsulfuration, transmethylation, and polyamine synthesis [1,2]. It is also widely used in treating clinical disorders such as liver disease, osteoarthritis, and depressive syndromes [3,4,5]. However, due to the high price of SAM, its wide application in the field of medicine has been limited. Because of the increasing medical demand for SAM, numerous attempts have been made to develop an efficient and low-cost means of producing SAM [6,7,8,9].

Currently, SAM is prepared industrially by high cell-density cultures of *Saccharomyces cerevisiae* or *Pichia pastoris*, which highly express MAT in the presence of exogenous l-methionine [9,10,11,12]. Genetic modification of yeast cells and the optimization of fermentation conditions are the two primary approaches to improving the production of SAM [13,14,15,16,17,18]. Zhao et al. integrated endogenous MAT into an industrial strain to improve SAM accumulation. The results showed that the recombinant yeast exhibited higher MAT activity and enhanced its SAM biosynthesis. With a fed-batch strategy in a 15 L bioreactor, 8.81 g/L of SAM was achieved after 52 h fermentation [9,11]. Additionally, the addition of l-methionine in the medium is required for the enhancement of both SAM production as well as cell growth. Chen et al. showed that the elevation of intracellular acetyl-CoA levels improved the intracellular methionine availability, which enhanced SAM accumulation (6.06 g/L) in yeast [13]. However, there are some obvious drawbacks to producing SAM via yeast fermentation, including a long fermentation period (48–120 h), high energy consumption, low SAM content and yield (~10 g/L medium), and a low conversion rate of l-methionine. Furthermore, the extraction and purification of SAM from yeast cells is complicated and environmentally unfriendly, since the yeast cells must be disrupted by first using a large amount of perchloric acid and then separating the SAM from the many other cell components [13].

In contrast to SAM production by yeast fermentation, the in vitro biosynthesis of SAM catalyzed by immobilized enzymes or immobilized whole-cells may be a more promising method due to a short reaction time (~5 h), a high substrate conversion rate (≥95%), a simple purification procedure, and environmental compatibility [6,7,19,20]. Whole cell lysates that contain the recombinant *Escherichia coli* (*E. coli*) MAT have already been used to synthesize SAM on the 30-mM scale [6,20]. A previous report from Luo et al. also showed that the recombinant yeast MAT expressed in *Pichia pastoris* had been employed to catalyze the synthesis of SAM [7]. However, the drawbacks of these free enzymes—including poor operational stability, high cost, and challenges in recovery and reuse—have limited industrial applications in the synthesis of SAM.

At present, there is little literature on the in vitro enzymatic synthesis of SAM using whole-cells highly expressing MATs as a source of biocatalysts. The biosynthesis of SAM using immobilized *E. coli* cells with high MAT activity may be advantageous by helping to avoid the purification of the enzyme from cells, simplifying the product purification process, increasing the stability of the enzymes, and reducing the cost of the biocatalyst [19]. Additionally, the immobilization of microbial cells using magnetic nanoparticles as an efficient immobilization method has been widely used for the biotransformation and biosynthesis of biomolecules [21,22,23,24,25,26]. The use of this immobilization method for biosynthesis is advantageous over traditional immobilization methods, in that it helps to increase the stability of the biocatalyst, reduce the mass transfer resistance, and facilitates its recovery and reuse. Therefore, the exploitation of magnetically immobilized cells highly expressing MAT in the biosynthesis of SAM appears to be a very promising approach.

In addition, choosing a suitable MAT enzyme has a pivotal role in the enzymatic synthesis of SAM. Compared with the MAT isoenzymes derived from other microorganisms (*Saccharomyces cerevisiae*, *Bacillus subtilis*, etc.) or from animal tissues [7,27,28,29], the MAT enzyme from *E. coli* has a number of advantages including high specific activity, a low *K*_m_ value, and highly efficient expression in *E. coli* [6,30]. Whole cell lysates of recombinant *E. coli* harboring the MAT gene have been employed to synthesize SAM [6,20]. However, the addition of a high concentration of sodium *p*-toluenesulfonate to the reaction mixture was required to relieve the product inhibition of the *E. coli* MAT enzyme. Therefore, it would be of great value to develop an engineered *E. coli* MAT variant with significantly reduced product inhibition in the biosynthesis of SAM.

In this study, a simple and efficient method for the enzymatic synthesis of SAM was developed by employing magnetically immobilized *E. coli* cells that highly expressed an engineered *E. coli* MAT variant with reduced product inhibition. Based on the crystal structure of *E. coli* MAT, the I303 residue was replaced with the less voluminous residue valine by site-directed mutagenesis. The generated I303V MAT variant significantly reduced the production inhibition. In addition, the characterization and recycling of the magnetically immobilized cells for SAM biosynthesis were also evaluated. Furthermore, we successfully used the magnetically immobilized cells to catalyze the production of SAM on the 40-mM scale.

## 2. Results and Discussion

### 2.1. Purification and Properties of Recombinant Wild-Type and I303V MAT

**Figure 1:**
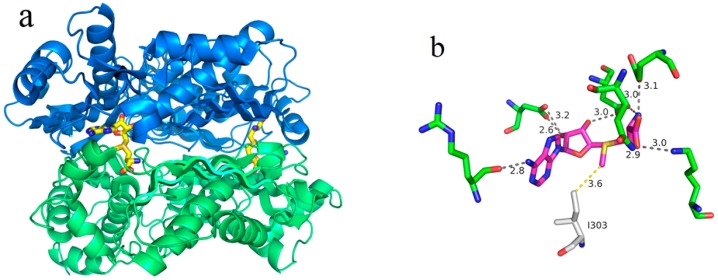
The 3D structure of the methionine adenosyltransferase (MAT) derived from *E. coli* (PDB: 1RG9). (**a**) The dimeric MAT with S-adenosylmethionine (SAM) bound between two monomer units; (**b**) The binding site of the product SAM (purple carbon atoms) in the active site of MAT. The interaction between the methyl group of SAM and the isoleucine I303 was hypothesized to affect the dissociation of SAM in the active center of the enzyme. Thus, isoleucine I303 became the target of site-directed mutagenesis.

The production of SAM using the wild-type *E. coli* MAT was not feasible due to the aforementioned production inhibition [20]. Therefore, engineered variants of MAT with significantly reduced product inhibition have been developed. In a recent study, Dippe et al. reported that an amino acid residue in the active center of the SAM synthase from *Bacillus subtilis* which directly interacts with the methyl group of SAM is the major steric hindrance acting upon the substrate into the active site of the enzyme [31]. We speculated that this interaction likely hinders the release of SAM from the active site of the enzyme, leading to the observed production inhibition. Based on this information, we next analyzed the tertiary structure of the *E. coli* MAT [32]. Figure 1a shows the complex structure of the MAT enzyme with its product SAM. A detailed examination of the active site indicated that the interaction between isoleucine I303 and the methyl group of SAM may contribute to product inhibition (Figure 1b). Therefore, the I303 residue was substituted with the less voluminous residue valine. Next, the expression and purification of the wild-type and I303V MAT proteins were carried out.

**Figure 2:**
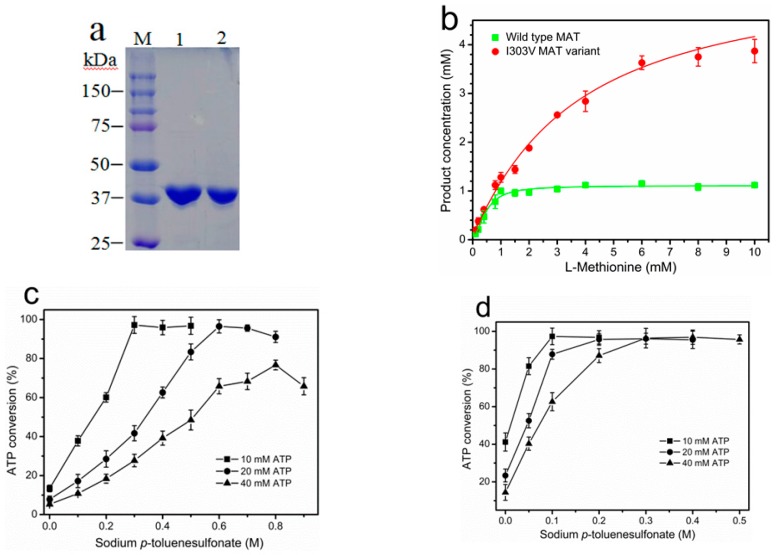
Conversion rates of adenosine triphosphate (ATP) catalyzed by the purified wild-type MAT and its variant I303V MAT. (**a**) SDS-PAGE (12%) analysis of the purified wild-type and I303V MAT proteins. Lane M: protein molecular weight marker. Lane 1: The purified wild-type MAT. Lane 2: The purified I303V MAT; (**b**) Production of SAM catalyzed by the wild-type and I303V MAT proteins as a function of the substrate concentration. The reaction mixtures, which contained 10 mM ATP and varying concentrations of l-methionine (0.1–10 mM), were incubated for 5 h at 37 °C in the presence of excess enzyme. The reaction yield was determined by measuring the phosphate that was released from the substrate ATP during the reaction. Curves were fitted by using the logistic model; (**c,d**) Effect of varying concentrations of sodium *p*-toluenesulfonate on the ATP conversion rate using wild-type MAT (**c**) and I303V MAT (**d**) in the presence of a 1.3-fold molar excess of l-methionine after a 6-h incubation at 37 °C, pH 7.0. The consumption of ATP and the production of SAM were determined by HPLC analysis.

The high expression of the recombinant wild-type MAT (~180 mg/L medium) and I303V MAT (~120 mg/L medium) in shake flask cultures was achieved. The expression level of the *E. coli* MAT was much higher than when the MAT from *S. cerevisiae* or *B. subtilis* is used [7,27]. Subsequently, both the wild-type and I303V MAT enzymes were purified by a single metal-affinity chromatography step to a homogeneous single band on SDS-PAGE (Figure 2a). In comparison with the low specific activity of the yeast MAT (~0.4 U/mg) [7], the specific activity of recombinant wild-type MAT and its variant I303V MAT was 3.6 U/mg and 14.2 U/mg, respectively. The recombinant I303V MAT had a four-fold increase in specific activity compared to the recombinant wild-type MAT. The *K*_m_ values for l-Met and ATP (*K*_m_^l-Met^ = 0.14 mM; *K*_m_^ATP^ = 0.38 mM) were slightly less than those of the wild-type MAT (*K*_m_^l-Met^ = 0.18 mM; *K*_m_^ATP^ = 0.46 mM). The total activities of the purified wild-type and I303V MAT enzymes obtained from 3-L cultures were ~1900 U and ~5100 U, respectively.

### 2.2. Product Inhibition of Wild-Type and I303V MAT

Next, the product inhibition of the purified wild-type and I303V MAT was evaluated. Both enzymes were employed to synthesize SAM in the presence of varying concentrations of l-methionine (0.1–10 mM) and 10 mM ATP. In line with a previous report, the wild-type MAT is subject to strong product inhibition when the concentration of SAM production is greater than 1 mM in reaction mixtures [20]. Expectedly, the product inhibition of I303V MAT was significantly reduced, and the product concentration reached approximately 4 mM in the presence of 10 mM ATP and l-methionine (Figure 2b). Moreover, enzymatic synthesis of SAM catalyzed by the wild-type and I303V MAT was performed on the 10–40 mM scale. As previously reported, the addition of a high concentration of sodium *p*-toluenesulfonate (0.6 M) to a 20-mM scale incubation was required to overcome the product inhibition of wild-type MAT [20]. However, only 75% of ATP conversion rate was reached, even though in the presence of 0.8 M sodium *p*-toluenesulfonate on a 40-mM scale (Figure 2c). Compared with the wild-type MAT, as little as 0.3 M sodium *p*-toluenesulfonate was required to completely overcome the product inhibition of I303V MAT on a 40-mM scale incubation (Figure 2d). These results indicated that the I303V MAT from *E. coli* would be a potential application for enzymatically producing SAM on an industrial scale.

### 2.3. Fermentation of Recombinant E. coli Expressing I303V MAT

**Figure 3:**
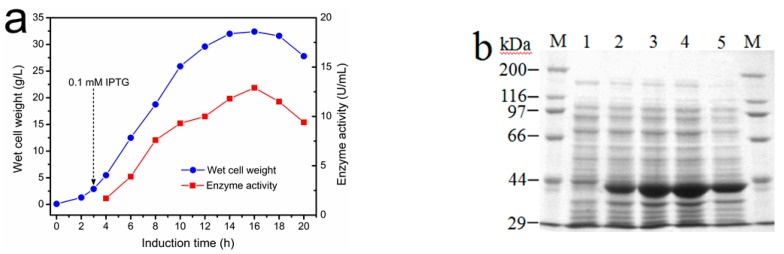
Expression of recombinant I303V MAT in a 10-L bioreactor. (**a**) Effect of induction time on cell biomass (g wet cell weight/L) and the activity of recombinant I303V MAT. The recombinant *E. coli* cells were induced by the addition of IPTG (isopropyl β-d-1-thiogalactopyranoside) to a final concentration of 0.1 mM; (**b**) SDS-PAGE (10%) analysis of the expression of the recombinant I303V MAT. Lane M: Molecular weight marker. Lane 1: Total protein of recombinant *E. coli* before IPTG induction. Lanes 2–5: Total protein of recombinant *E. coli* induced with IPTG after 5, 9, 13, and 17 h, respectively.

To obtain a large amount of the biocatalyst for SAM synthesis, the recombinant *E. coli* harboring the I303V MAT gene was cultured in a 10-L bioreactor. The *E. coil* strains were grown in TB medium to an OD_600_ of ~1.5 at 30 °C (~3 h), at which point IPTG (isopropyl β-d-1-thiogalactopyranoside) was added to a final concentration of 0.1 mM. The effect of induction time of IPTG on the volumetric activity of the recombinant enzyme is shown in Figure 3a. With an increase in induction time of IPTG, the activity of the recombinant I303V MAT rapidly increased. The highest activity was approximately 13,000 U/L medium after a 16-h culture, much higher than the highest activity attained using recombinant yeast MAT expressed in *P. pastoris* after a 160-h induction (80 U/L medium in a bioreactor) [7]. Changes in the protein expression levels of I303V MAT were determined by SDS-PAGE (Figure 3b). The volumetric activity of recombinant I303V MAT increased approximately 160-fold compared with the yeast MAT expressed in *P. pastoris* [7]. The *E. coli* cells were then collected and used to prepare the immobilized cells.

### 2.4. Permeabilization of Free Whole-Cells of E. coli

**Figure 4:**
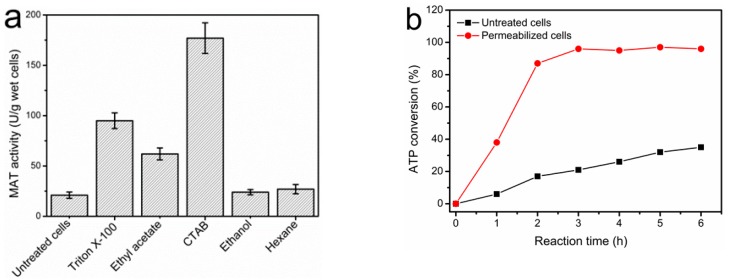
Permeabilization of free whole*-*cells *of E. coli* and synthesis of SAM catalyzed by the untreated and permeabilized cells. (**a**) Cell-bound I303V MAT activity for *E. coli* cells permeabilized with different detergents (0.1% *w*/*v*) or organic solvents. Treatment was carried out for 30 min with 10 mL/g wet cell weight; (**b**) Conversion rate of the ATP substrate catalyzed by untreated and permeabilized *E. coli* cells as a function of time. Reactions were performed in a 100-mL volume and included: 40 mM ATP, 50 mM l-methionine (l-Met), 50 mM K_2_SO_4_, 100 mM MgSO_4_, 0.3 M sodium *p*-toluenesulfonate, 100 mM Tris-HCl, and 1 g of cells at 37 °C, pH 7.0. The consumption of ATP and the production of SAM were determined by HPLC analysis, and the MAT activity was calculated according to the production of SAM.

The use of whole-cells for biosynthesis and biotransformation circumvents the need to purify intracellular enzymes and provides a more favorable environment for the enzymes to function. Furthermore, the immobilization of whole-cells can facilitate product purification and allows for the recycling of the biocatalysts, thus lowering the cost of production. However, this method also suffers from lower activity compared to using free enzymes because of the limited diffusion of the reaction substrates or products through the cell membrane [33]. Treatment of the cell with chemical agents will permeabilize the membrane, thereby alleviating this problem. In this study, a variety of solvents and detergents were used to permeabilize the free whole-cells of *E. coli* expressing the I303V MAT enzyme. The detergent CTAB (hexadecyl trimethyl ammonium bromide) significantly increased the cell-bound I303V MAT activity by 8.4-fold, while other agents (e.g., ethyl acetate and Triton X-100) produced a moderate increase in MAT activity (Figure 4a). In many previous studies, CTAB has been identified as a potent permeabilizing agent for increasing the activity of recombinant enzymes expressed in *E. coli* [34,35,36]. The permeabilizing conditions were optimized with respect to CTAB concentration, volume, and treatment time to further enhance the I303V MAT activity. The activity of permeabilized *E. coli* cells reached 177 U/g wet cell weight upon treatment with 0.1% (*w*/*v*) CTAB for 30 min, while the activity of untreated cells was 21 U/g wet cell weight. Both the permeabilized cells and untreated cells were used to catalyze the biosynthesis of SAM. After a 3-h incubation, the substrate ATP was completely converted using the permeabilized cells as a biocatalyst, while only a 30% conversion rate was achieved after a 6-h incubation using the untreated *E. coli* cells (Figure 4b).

### 2.5. Preparation of Magnetically Immobilized Cells

The use of whole-cells as a biocatalyst is a promising strategy for avoiding some drawbacks associated with the use of pure enzymes—especially their high cost [19]. The use of free cells is also challenging, since cell lysis can also occur under the reaction conditions. However, cell immobilization has been employed to increase the catalytic potential of enzymes by extending their lifetimes under the reaction conditions. Besides, immobilized cells maintain their biocatalytic performance for several reaction cycles. Furthermore, the use of magnetically immobilized cells for biosynthesis is advantageous over traditional immobilization methods in that it helps to reduce the mass transfer resistance and facilitates its recovery and reuse [21,22,23,24].

**Figure 5:**
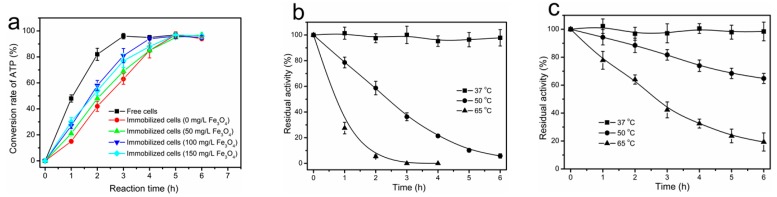
Effect of the concentration of Fe_3_O_4_ nanoparticles on the activity of immobilized cells, and the thermal stability of free and magnetically immobilized cells; (**a**) Biosynthesis of SAM by magnetically immobilized cells prepared by the addition of different concentration of Fe_3_O_4_ nanoparticles (50–150 mg/L), nonmagnetically immobilized cells, and free cells. The reaction mixtures contained: 40 mM ATP, 50 mM l-methionine, 50 mM K_2_SO_4_, 100 mM MgSO_4_, 0.3 M sodium *p*-toluenesulfonate, 100 mM Tris-HCl (pH 7.0), and the equivalent amount of biocatalyst for free or immobilized cells, at 37 °C. The conversion rate of ATP was determined by HPLC analysis; (**b**,**c**) The thermal stability of free and magnetically immobilized cells. Percent enzymatic activity remaining as a function of time for free (**b**); and magnetically immobilized cells (**c**). Assay conditions: 50 mM Tris-HCl buffer, pH 7.5. Initial activities were defined as 100%. The residual activity of free and immobilized cells was measured using the spectrophotometric assay. Enzyme activity was calculated according to the phosphate concentration that was released from the substrate ATP during the reaction.

In this study, the permeabilized cells were immobilized with gellan gum gel entrapment in the presence of Fe_3_O_4_ nanoparticles. The effect of different concentrations of Fe_3_O_4_ nanoparticles (50, 100, and 150 mg/L) on the activity of the immobilized cells was studied. The biosynthesis of SAM catalyzed by free cells, nonmagnetically immobilized cells, and magnetically immobilized cells was also investigated. As shown in Figure 5a, the enzyme activity of both magnetically immobilized cells and nonmagnetically immobilized cells was significantly decreased compared to the free cells. However, the magnetically immobilized cells showed a higher activity than the nonmagnetically immobilized cells. Among the assayed concentrations of magnetic Fe_3_O_4_ nanoparticles, the enzyme activity for SAM synthesis was highest at a concentration of 100 mg/L, where 40 mM of ATP was completely converted to SAM in 4 h, while the equivalent amount of ATP could be completely converted in 5 h by nonmagnetically immobilized cells. These results revealed that SAM biosynthetic activity of I303V MAT in the immobilized *E. coli* cells was significantly enhanced by the addition of different concentrations of magnetic Fe_3_O_4_ nanoparticles—especially the concentration of 100 mg/L. The increase in the activity of magnetically immobilized cells may be due to the fact that the existence of Fe_3_O_4_ nanoparticles might increase the bioavailable concentration in the inner spaces of the gel beads, and then reduce the mass transfer problems [21,22,37].

We next investigated the effect of the quantity of *E. coli* cells (10–50 g/L support) on the immobilization procedure. In each experiment, the different amounts of *E. coli* cells were mixed with a certain volume of gellan gum gel (1%, *w*/*v*) in the presence of 100 mg/L Fe_3_O_4_ nanoparticles. Table 1 showed that the activity of immobilized cells increased rapidly as loading of *E. coli* cells increased, and the activity leveled off at 30 g/L support. The activity recovery is inversely related to the number of cells that were loaded. For example, increasing cell loading from 10 to 50 g/L support resulted in decreasing the activity recovery from 86.4 to 45.8%, possibly due to packing of the cells in the support that could limit the access of substrates and thus reduce enzyme activity. It is generally acknowledged that the activity recovery of immobilization processes decreases when loading exceeds a certain value [38,39]. In this study, the maximum activity of the immobilized cells that was attained was 4152.4 U/L support, and the corresponding activity recovery was 78.2%.

In addition, the kinetics of the magnetically immobilized cells were investigated by using varying concentrations of the l-methionine and ATP substrates. The kinetic constant *K*_m_ was obtained according to the double reciprocal plot, and the values of *K*_m_^ATP^ and *K*_m_^l-Met^ were 0.53 mM and 0.34 mM, respectively. These values were significantly lower than the *K*_m_^ATP^ = 0.86 mM and *K*_m_^l-Met^ = 0.55 mM values obtained for nonmagnetically immobilized cells. The decrease in *K*_m_ values may be due to a reduction or elimination of mass transfer problems [21,37].

### 2.6. Thermal Inactivation of Free and Immobilized Cells

Due to the poor thermal stability of the MAT enzymes derived from *E. coli* and *S. cerevisiae*, the use of free whole-cells expressing these enzymes is not feasible to catalyze the synthesis of SAM at an industrial scale. For example, after the preincubation of the recombinant yeast MAT at 50 °C for 2 h, the residual activity was less than 10% [7,28]. After incubation of free *E. coli* whole-cells at 50 °C for 2 h and 5 h, the residual activities were 59% and 10%, respectively (Figure 5b). Thus, the primary goal of this study was to improve the biocatalyst stability and develop an immobilized whole-cell that is capable of synthesizing SAM for extended periods of time. As a result, the thermal stabilities of the free cells and magnetically immobilized cells were compared over a temperature range of 37–65 °C. In both cases, the activity of the I303V MAT enzyme was stable at 37 °C for a 6 h incubation (Figure 5b,c). However, over a temperature range of 50–65 °C, the stability of the immobilized cells was much higher than that of the free cells. Plots of the natural log of the relative I303V MAT activities as a function of time showed first-order kinetics for both free cells and immobilized cells and were used to evaluate deactivation constants (*k*_d_) and half-life values (*t*_1/2_). For the free cells, the estimated parameters were *k*_d_ = 0.487/h and *t*_1/2_ = 1.4 h at 50 °C, while for the immobilized cells the estimated parameters were *k*_d_ = 0.0759/h and *t*_1/2_ = 9.1 h at 50 °C. In general, the stability of immobilized whole-cells on gellan gum entrapment was improved compared to free cells (Figure 5b,c), suggesting that cell immobilization with gellan gum entrapment can enhance the biocatalyst stability, making them less prone to enzyme denaturation. According to the obtained *t*_1/2_ values, it can be concluded that the magnetically immobilized cells were 6.5-fold more stable than the free cells, suggesting that cell immobilization on gellan gum may be beneficial to the enzyme activity of cells.

### 2.7. Reusability of Magnetically Immobilized Cells for SAM Production

**Figure 6:**
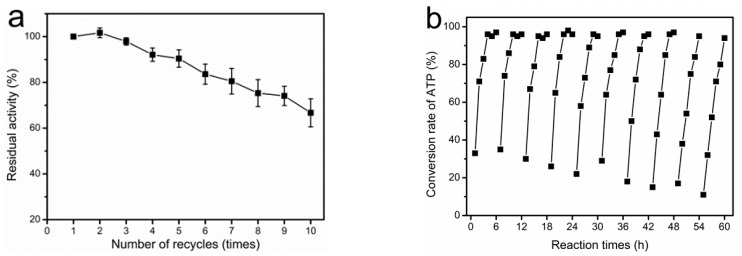
Reuse of magnetically immobilized cells for SAM biosynthesis. (**a**) The effect of recycling on the activity of magnetically immobilized cells. The residual activity was measured using the spectrophotometric assay. Enzyme activity was calculated according to the phosphate concentration that was released from the substrate ATP during the reaction; (**b**) Production of SAM with magnetically immobilized cells in 10 repeated batches. The reaction was performed in 100 mM Tris-HCl buffer containing 40 mM ATP, 50 mM l-methionine, 50 mM K_2_SO_4_, 100 mM MgSO_4_, and 0.3 M sodium *p*-toluenesulfonate, pH 7.0 with a 6-h incubation at 37 °C. The consumption of ATP and the production of SAM were determined by HPLC analysis, and the conversion rate of ATP was calculated.

The reusability of the immobilized cells is an important factor that determines the effectiveness of SAM production in an industrial process. In the current study, the operational stability of the magnetically immobilized cells was evaluated in a repeated batch process. The magnetically immobilized cells retained 90% of their initial activity after five reuses and 67% after ten reuses (Figure 6a). No leakage of MAT activity from the support was observed during repeated batch process, suggesting that the deactivation is essentially due to the activity loss of the enzyme, and not to its desorption from the support. In contrast, the retention of activity of the immobilized yeast MAT after three reuses was only ~10% of its initial activity [7]. Furthermore, the magnetically immobilized cells could be effectively re-used 10 times to produce SAM on a 40-mM scale, with a high conversion rate of the ATP substrate (≥95%). From the first to the fourth cycle, the 40 mM ATP was completely converted after 4 h. A decrease in enzyme activity from the fifth to the tenth cycle resulted in it taking 5–6 h to completely covert the 40 mM ATP (Figure 6b). Compared to the enzymatic production of SAM reported in other studies [6,7,20,31], our results showed a significant improvement on the production of SAM and had a short reaction time (6 h) by using magnetically immobilized cells.

## 3. Materials and Methods

### 3.1. Materials

l-Methionine (l-Met), isopropyl β-d-1-thiogalactopyranoside (IPTG), and kanamycin were obtained from Amresco (Solon, OH, USA); adenosine triphosphate (ATP) was purchased from Kaiping Genuine Biochemical Pharmaceutical Co., Ltd. (Jiangmen, China); and S-adenosylmethionine and gellan gum were obtained from Sigma-Aldrich (St. Louis, MO, USA). Magnetic Fe_3_O_4_ nanoparticles (diameter 20 nm, 99%) were purchased from Xi'an Ruixi Biological Technology Co., Ltd. (Xi'an, China). DNA polymerase, restriction enzymes, and T4 DNA ligase were obtained from TaKaRa (Tokyo, Japan). *E. coli* DH5α was used as a host strain for DNA manipulation, and *E. coli* BL21 (DE3) was used for MAT and I303V MAT gene expression. The vector pET-28a (+) was purchased from Novagen (Madison, WI, USA). Yeast extract and peptone were obtained from Oxoid (Hampshire, UK). Ni Sepharose 6 Fast Flow resin and HiTrap desalting columns were purchased from GE Healthcare (Little Chalfont, Buckinghamshire, UK). All other commercially available chemicals were of analytical grade.

### 3.2. Preparation of Recombinant, Wild-Type, and I303V MAT

The methionine adenosyltransferase (MAT) gene from *E. coli* was amplified using the genome of *E. coli* DH5α as template DNA with the following primers: 5′-ACGTGAATTCATGGCAAAACACCTTTTTAC-3′and 5′-ATGTAAGCTTTTACTTCAGACCGGCAGCATC-3′ (the *Eco*RI and *Hin*dIII restriction sites are underlined). The MAT gene was cloned into the pET-28a (+) vector to generate the recombinant plasmid pET-28a-MAT. To construct an I303V MAT variant, the wild-type MAT gene was modified by site-directed mutagenesis. The recombinant plasmids were then transformed into *E. coli* BL21 (DE3) for expression of the wild-type and I303V MAT proteins. The recombinant strains were grown in LB medium containing 50 mg/L kanamycin to an optical density (OD_600_) of 0.4–0.6, then IPTG was added to a final concentration of 0.1 mM. After incubation at 30 °C for 12–16 h, cells were collected by centrifugation at 6000 rpm for 10 min at 4 °C. Cell pellets obtained from a 3-L cultures were resuspended in 300 mL of phosphate-buffered saline (PBS). The cells were disrupted by sonication, and the supernatants were obtained by centrifugation at 12,000 rpm for 30 min at 4 °C. The recombinant wild-type and I303V MAT proteins were purified using a HiTrap chelating nickel column (elution buffer: 50 mM sodium phosphate, 300 mM NaCl, 300 mM imidazole, pH 7.4), followed by a desalting column using gel filtration buffer (25 mM Tris-HCl, pH 7.4). Fractions containing the wild-type or I303V MAT were pooled, and the protein concentrations were measured by the Coomassie Blue method. The aliquoted proteins were stored at −80 °C.

Because a large amount of the recombinant I303V MAT enzyme was required for the biosynthesis of SAM, the fermentation of the recombinant *E. coli* was carried out in a 10-L bioreactor (Baoxing, Shanghai, China) with 6 L of TB medium (12 g/L peptone, 24 g/L yeast extract, 17 mM KH_2_PO_4_, 72 mM K_2_HPO_4_, and 4 mL/L glycerol) that was supplemented with 50 mM kanamycin. The inoculum was grown at 30 °C in a 2-L shake flask to an OD_600_ of 0.4–0.6 in LB medium, and then 300 mL of inoculum was added to the bioreactor. The *E. coil* strains were grown in TB medium to an OD_600_ of ~1.5 at 30 °C, at which point IPTG was added to a final concentration of 0.1 mM. Dissolved oxygen was maintained at more than 20% saturation by adjusting the stirring rate. The fermentation process was carried out in batch mode until the glycerol was completely consumed. The biomass was determined by measuring the wet cell weight and the OD_600_ of the cell culture medium. The recombinant proteins were analyzed with a 12% SDS-PAGE gel under reducing conditions.

### 3.3. Permeabilization of E. coli cells

A previously reported method was used with some modifications to permeabilize cells [36]. *E. coli* cells were suspended in 50 mM phosphate-buffered saline, pH 7.5 (1 g wet cell weight/10 mL), and the different detergents or organic solvents were added to a final concentration of 0.1% (*w*/*v*). The cell suspensions were then allowed to stir gently on a rotary shaker at 25 °C for 30 min. Treated cells were recovered by centrifugation at 6000 rpm and 4 °C for 30 min, followed by washing the cells 2–3 times with 50 mM Tris-HCl buffer (pH 7.5) to remove CTAB. Then, the permeabilized cells were collected and used to prepare the immobilized cells.

### 3.4. Preparation of Nonmagnetically and Magnetically Immobilized Cells

The immobilized *E. coli* cells were prepared according to a previously report [37]. The permeabilized cells were resuspended in 50 mM Tris-HCl buffer (pH 7.5), and then the suspension of *E. coli* cells and the gellan gum (1%, *w*/*v*) were mixed at a ratio of wet cell weight to dry gellan gum of 1–5 (*w*/*w*). Nonmagnetically immobilized cells were prepared by extruding the mixture through a syringe into 0.2 M CaCl_2_ and letting it solidify for 2 h. For the preparation of magnetically immobilized cells, a different quantity of Fe_3_O_4_ nanoparticles was added to the mixture of gellan gel and cell suspension, and the subsequent procedures were the same as those used for the nonmagnetically immobilized cells. The average bead diameter was 2 mm. The immobilized whole-cells were then used for SAM production. The activity of the immobilized cells was determined by HPLC analysis according to the production of SAM, and activity recovery is presented as the percentage of the activity of the immobilized cells compared to the activity of the free whole-cells, which was taken as 100%.

### 3.5. Spectrophotometric Assay for MAT Activity

The activities of the purified wild-type and I303V MAT proteins were determined in a microplate at 37 °C according to a previous report [31]. Briefly, the 50-μL assay mixture contained 0–10 mM l-Met, 10 mM ATP, 50 mM K_2_SO_4_, 20 mM MgSO_4_, and 100 mM Tris-HCl, pH 8.0. The assay mixture was incubated at 37 °C for 5 min, and the reaction was initiated by the addition of the recombinant MAT or I303V MAT enzyme. After an incubation for 30 min, the concentration of phosphate that had been released from ATP was measured. In total, 100 μL of malachite green-molybdate solution was added, and the absorbance at 620 nm was measured after a 5 min incubation. A standard solution of sodium phosphate (0–75 μM Na_2_HPO_4_ and Na_2_H_2_P_2_O_7_ in 10 mM ATP) was used to calculate the phosphate concentration. The enzyme activity was calculated based on a linear fit to the phosphate concentration. One unit (U) of enzyme activity was defined as the amount of MAT that catalyzes the formation of 1 μM phosphate per minute at 37 °C, pH 8.0. For the kinetic analysis; the reaction system was the same as for the enzyme activity determination, except the concentration of l-Met or ATP substrates was varied from 0.01–1.0 mM. When the MAT activities of free and immobilized cells were measured, the whole-cells of *E. coli* which do not express the recombinant MAT enzyme were set as negative control groups.

### 3.6. High-Performance Liquid Chromatography (HPLC) Analysis

The enzymatic synthesis of SAM from l-Met and ATP was catalyzed by free MAT enzyme or whole-cells in the presence of a specific concentration of *p*-toluenesulfonate. The supernatants were analyzed by HPLC using a C18 column (4.6 mm × 250 mm, 5 μM), monitoring the absorption of the ATP substrate and the SAM product at 254 nm. The mobile phase consisted of 1% acetic acid (*v*/*v*), 15% acetonitrile (*v*/*v*), and 10 mM sodium 1-hexanesulfonate, using a flow rate of 1 mL/min. A peak area analysis was performed based on the standard calibration curves of ATP and SAM. One unit (U) of enzyme activity was defined as the amount of MAT that catalyzes the formation of 1 μM SAM per minute at 37 °C, pH 8.0.

### 3.7. Thermal Stability of the Free and Magnetically Immobilized Cells

Thermal stability assays were performed at three different temperatures (37, 50, and 65 °C) in 50 mM Tris-HCl, pH 7.5. The free cells (1 g wet cell weight/10 mL buffer) were incubated for various time periods at different temperatures. Next, the residual I303V MAT activities were measured using a spectrophotometric assay. Enzyme activity was calculated according to the phosphate concentration that was released from the substrate ATP during the reaction. For the magnetically immobilized cells, the same amount of biocatalyst (equivalent to 1 g wet cell weight in 10 mL of buffer) was incubated at 37–65 °C in 50 mM Tris-HCl buffer, pH 7.5. After the different incubation times, the residual I303V MAT activities were determined according to the method described above. Relative activity is presented as the percentage of the MAT activity that was retained compared to the activity that was exhibited before the incubation, which was set as 100%. Data from experiments are expressed as the mean ± SD.

### 3.8. Reusability of the Magnetically Immobilized Cells for SAM Biosynthesis

A study of the production of SAM by reused magnetically immobilized cells was carried out. The reaction mixture consisted of 40 mM ATP, 50 mM l-Met, 50 mM K_2_SO_4_, 100 mM MgSO_4_, 0.3 M sodium *p*-toluenesulfonate, and 100 mM Tris-HCl, pH 7.0. After the addition of the magnetically immobilized cells (20%, *w*/*v*), the mixture was incubated for 6 h at 37 °C. The leaked MAT activity of the immobilized cells in the reaction mixture was measured using the spectrophotometric assay described above. In the recycling experiments, the magnetically immobilized cells were collected from the reaction mixture by filtration and were washed with 100 mM Tris-HCl, pH 7.0. The immobilized cells were reused for the following batch. The consumption of ATP and the production of SAM in the reaction mixture were determined by HPLC analysis, and the conversion rate of ATP was calculated. The residual activity of the magnetically immobilized cells was determined by the use of a spectrophotometric assay. Enzyme activity was calculated according to the phosphate concentration that was released from the substrate ATP during the reaction. The enzyme activity of the immobilized cells in the first batch was set as 100%. The residual activity after each use was calculated by dividing the activity after each batch by the activity in the first batch and multiplying by 100.

## 4. Conclusions

In this study, an engineered variant of the *E. coli* MAT enzyme (I303V MAT) significantly reduced the product inhibition during SAM biosynthesis and was highly expressed (~900 mg/L) in a 10-L bioreactor. The specific activity of I303V MAT had a four-fold increase compared to the recombinant wild-type MAT. To reduce the mass transfer resistance during the process of SAM biosynthesis, the recombinant *E. coli* cells were permeabilized using 0.1% CTAB. Then, the permeabilized cells were immobilized using gellan gum gel as support in the presence of 100 mg/L Fe_3_O_4_ nanoparticles, and the highest activity (4152.4 U/L support) was obtained, with a 78.2% of the activity recovery. The obtained magnetically immobilized cells had lower *K*_m_ values than nonmagnetically immobilized cells. Furthermore, the recycling experiments demonstrated that the residual activity of the magnetically immobilized cells retained 67% of their initial activity after 10 reuses, with ATP substrate conversion rates ≥95% for all the 40-mM-scale reactions. Based on these findings, magnetically immobilized cells harboring the I303V MAT gene should be a promising biocatalyst used in the production of SAM.

## Figures and Tables

**Table 1:** Influence of the quantity of *E. coli* cells on the immobilization procedure. The suspension of *E. coli* cells and the gellan gum (1%, *w*/*v*) were mixed at a ratio of wet cell weight to dry gellan gum of 1–5 (*w*/*w*) in 50 mM Tris-HCl buffer, pH 7.5. The activity of permeabilized cells is 177 U/g wet cell weight.

Cell Loading (g/L Support)	Activity of Immobilized Cells (U/L Support)	Activity Recovery (%)
10	1529.3	86.4
20	2906.3	82.1
30	4152.4	78.2
40	3957.7	55.9
50	4053.5	45.8
